# Editorial: Mechanism‐specific developmental windows of opportunity in the prevention of youth psychopathology

**DOI:** 10.1111/jcpp.70203

**Published:** 2026-07-13

**Authors:** Katie L. Burkhouse, Joan L. Luby

**Affiliations:** ^1^ Department of Psychology The Pennsylvania State University University Park PA USA; ^2^ Department of Psychiatry, School of Medicine Washington University in St. Louis St. Louis MO USA

**Keywords:** Precision prevention, developmental timing, sensitive periods, mechanism‐based intervention and neurodevelopment

## Abstract

Mechanism‐based prevention has transformed developmental psychopathology by shifting the focus from whether interventions work to understanding how they work through the modification of neurobehavioral processes underlying risk. We argue that this framework remains incomplete without equal consideration of *when* during development interventions are delivered. Because neurobehavioral mechanisms emerge and mature along distinct developmental trajectories, interventions are likely to be most effective when they target mechanisms during periods of heightened developmental plasticity. We propose a framework of mechanism‐specific developmental windows of opportunity, in which the timing of intervention is considered alongside mechanistic target selection. Evidence from developmental neuroscience and prevention research suggests that interventions delivered during different developmental periods may capitalize on distinct forms of neurobehavioral plasticity, from broad foundational processes in infancy to more specialized systems that continue maturing throughout childhood and adolescence. We further highlight that environmental context, particularly early adversity, may shape the malleability of these systems and influence responsiveness to intervention. Integrating developmental timing into mechanism‐based prevention has the potential to improve intervention precision and alter trajectories of child and adolescent psychopathology.

Over the past two decades, prevention science has increasingly shifted from asking whether interventions work to understanding how they work. This transition has been driven, in part, by the National Institutes of Health's experimental therapeutics framework, which emphasizes identifying and testing specific mechanisms hypothesized to underlie psychopathology (Zucker et al., 2025). Rather than focusing solely on symptom reduction, this approach evaluates whether modifying targeted neurobehavioral processes that subserve social and emotional functioning alters risk trajectories. As a result, considerable progress has been made in identifying mechanisms that confer vulnerability to child and adolescent psychopathology, advancing the development and precision of mechanism‐focused preventive interventions.

However, identifying a mechanistic target is only one dimension of precision prevention. An equally important question is whether interventions are differentially effective depending on when in development they are delivered. This reflects the premise that neurobehavioral systems follow dynamic developmental trajectories that may constrain or enhance their capacity for change at different points in time. Here, we propose that developmental timing represents a critical, yet often overlooked, dimension of mechanism‐based prevention, such that interventions should be designed not only to target the appropriate mechanism, but also to capitalize on developmental periods during which that mechanism is most malleable and most likely to alter long‐term trajectories.

This perspective builds on the premise that neurobehavioral mechanisms do not emerge or mature uniformly across development. Rather, converging research suggests that different neurobehavioral systems exhibit distinct developmental trajectories characterized by periods of heightened plasticity. Consequently, different mechanisms are likely to possess distinct developmental windows of opportunity during which they are especially amenable to intervention. For example, early childhood has been identified as a particularly important period for shaping stress‐response systems and emotion regulation through caregiver‐child interactions (Gee, 2016), whereas middle childhood and adolescence may represent key windows for interventions targeting executive control as prefrontal systems mature and social‐affective processes, including sensitivity to peer evaluation and social reward, increase (Blakemore & Mills, 2014; Selemon, 2013). These developmental transitions may represent windows during which interventions can produce larger and more enduring effects. Rather than viewing developmental timing as a secondary design consideration, we suggest that it should be considered alongside mechanistic target selection when designing preventive interventions. Just as neurobehavioral mechanisms emerge and mature at different periods across development, their capacity to be experimentally altered may likewise be developmentally constrained. From this perspective, developmental windows may determine not only the magnitude of intervention effects, but also whether the targeted neurobehavioral mechanism is sufficiently developmentally malleable to be modified by intervention.

The importance of developmental timing and its impact on long‐term outcomes is well established in research on early adversity. Work on sensitive periods has shown that experiences such as deprivation, neglect, and chronic stress exert particularly pronounced effects when they occur during specific developmental windows, particularly early childhood (Gabard‐Durnam & McLaughlin, 2019). Extending this work, Eldeeb et al. (2026), in this issue, demonstrate that the timing of adversity differentially predicts childhood psychopathology globally, with distinct sensitive periods emerging for specific forms of adversity. Notably, the authors suggest that these findings may help inform the timing of future interventions, highlighting the growing recognition that *when* adversity occurs may be as important as the type of adversity. As sensitive periods are known to characterize the developmental impact of adversity, they can likewise help clarify opportunities for prevention. This perspective also suggests that mistimed intervention may limit efficacy. Intervening before a neurobehavioral mechanism has sufficiently emerged or well after it has been concretized may reduce opportunities for meaningful engagement, diminishing the potential to alter long‐term trajectories.

Despite growing recognition that developmental windows shape vulnerability to psychopathology, less attention has been devoted to empirically determining optimal developmental timing for preventive interventions. If the developmental modifiability of neurobehavioral mechanisms varies across childhood and adolescence, prevention efforts should align intervention delivery with periods during which targeted mechanisms are most responsive to change. Two recent examples illustrate how windows of development can inform mechanism‐based prevention across childhood. First, Luby et al. (2024) provide a demonstration of developmentally sensitive prevention in infancy that targets global and foundational aspects of development at the earliest possible point beginning at birth. Rather than targeting a single intervention component, the authors operationalized a constellation of modifiable environmental supports, including positive caregiving, cognitive stimulation, nutrition, sleep, and neighborhood safety, that collectively shape early neurodevelopmental context. Environmental supports in the first year of life predicted larger cortical gray matter volume, stronger cognitive performance, and fewer internalizing and externalizing symptoms in early childhood, even after accounting for prenatal social disadvantage. These findings underscore a key developmental principle consistent with the present framework: infancy may represent a period of heightened neurodevelopmental plasticity, during which relatively modest improvements in basic environmental input can shape multiple interrelated neural and behavioral systems.

If the developmental modifiability of neurobehavioral mechanisms varies across childhood and adolescence, prevention efforts should align intervention delivery with periods during which targeted mechanisms are most responsive to change.

Importantly, windows of opportunity for prevention are unlikely to be equivalent across development. During infancy, interventions may exert broad, system‐wide effects by shaping foundational neurodevelopmental processes that influence multiple downstream mechanisms. As children mature, however, prevention may increasingly benefit from targeting more specialized neurobehavioral systems that are undergoing continued development and become more directly linked to specific forms of psychopathology. Positive valence systems provide one such example, given their prolonged development across childhood and adolescence and their established role in depression risk (Kujawa & Burkhouse, 2017). To illustrate this principle, Burkhouse and colleagues (2023) developed and tested Family Promoting Positive Emotions (FPPE), a prevention program grounded in evidence that alterations in reward responsiveness predict depression risk in children of depressed mothers. FPPE is a dyadic preventive intervention designed to enhance positive affect within the mother–child relationship and strengthen reward responsiveness during middle childhood, a developmental period characterized by continued maturation of positive valence systems, increasing depression risk, and children's growing capacity to recognize, anticipate, and sustain complex positive emotional experiences within close relationships. The intervention emphasizes repeated opportunities for shared enjoyment, warmth, and rewarding social interaction in mother–child dyads, with the goal of strengthening child reward‐related functioning during ongoing neurodevelopmental plasticity. Emerging findings suggest that increasing these positive relational experiences in middle childhood can improve children's emotional functioning prior to the typical escalation in depression risk during adolescence. Together, these findings illustrate a central principle of developmentally informed prevention: the effectiveness of mechanistic interventions depends not only on targeting the appropriate neurobehavioral system but also on aligning intervention timing with periods of meaningful developmental change.

An additional cross‐cutting observation across these studies further underscores the importance of developmental context for mechanism‐based prevention: in both infancy and middle childhood, intervention effects were most pronounced for children exposed to higher levels of early adversity. In Luby et al. (2024), basic environmental enrichment during the first year of life showed particularly strong associations with socioemotional outcomes among children experiencing greater prenatal social disadvantage. Similarly, in the FPPE trial, effects of the intervention on changes in child clinical symptoms were most evident among high‐risk children of mothers living in neighborhoods characterized by greater deprivation (Yerger et al., 2026). Together, these findings suggest that developmental timing alone is unlikely to optimize intervention impact. Rather, the developmental malleability of neurobehavioral systems may itself vary as a function of environmental context, suggesting that experiences of adversity may shape the extent to which neurobehavioral systems remain responsive to subsequent environmental input. This view converges with developmental models suggesting that early adversity shapes the calibration of neurobehavioral systems, altering subsequent environmental sensitivity and learning from later experience (Boyce & Ellis, 2005; Frankenhuis & de Weerth, 2013).

Together, these examples support a broader conceptual framework in which intervention timing represents an essential dimension of mechanism‐based prevention. Rather than assuming a single developmental window during which prevention is universally most effective, the emerging evidence is more consistent with mechanism‐specific neurodevelopmental windows of opportunity, whereby distinct neural systems may be differentially amenable to intervention across development.

Moving forward, mechanism‐focused prevention studies should begin to address three interrelated questions. First, when does the target mechanism become developmentally influential for psychopathology? Second, during which developmental period is that mechanism most amenable to experimental engagement or change? Third, does intervening during that developmental window alter downstream developmental cascades more effectively than intervention delivered earlier or later? Addressing these questions will require longitudinal randomized controlled prevention trials spanning multiple developmental periods, repeated assessments of neurobehavioral mechanisms, and trials explicitly powered to evaluate developmental timing as a core design feature rather than a secondary moderator. Ultimately, precision prevention will depend not only on identifying the right mechanisms, but on engaging the right mechanisms at the right developmental period. Developmental timing should therefore be viewed not as a secondary consideration after intervention targets have been selected, but as a fundamental principle guiding the design, implementation, and evaluation of mechanism‐based interventions for the prevention of child and adolescent psychopathology. Aligning intervention targets with mechanism‐specific developmental windows of opportunity may enhance the durability of effects and improve translation of mechanistic insights into altered developmental trajectories.
